# A de novo 2.2 Mb recurrent 17q23.1q23.2 deletion unmasks novel putative regulatory non-coding SNVs associated with lethal lung hypoplasia and pulmonary hypertension: a case report

**DOI:** 10.1186/s12920-020-0701-6

**Published:** 2020-03-06

**Authors:** Justyna A. Karolak, Tomasz Gambin, Engela M. Honey, Tomas Slavik, Edwina Popek, Paweł Stankiewicz

**Affiliations:** 1 Department of Molecular & Human Genetics, Baylor College of Medicine, Houston, TX 77030 USA; 2 Chair and Department of Genetics and Pharmaceutical Microbiology, Poznan University of Medical Sciences, 60-781 Poznan, Poland; 3 Institute of Computer Science, Warsaw University of Technology, 00-665 Warsaw, Poland; 4 Department of Biochemistry, Genetics and Microbiology, Faculty of Natural and Agricultural Science, University of Pretoria, Pretoria, South Africa; 5 Ampath Pathology Laboratories, and Department of Anatomical Pathology, University of Pretoria, Pretoria, South Africa; 6 Department of Pathology and Immunology, Baylor College of Medicine, Houston, TX 77030 USA

**Keywords:** Multi-locus genomic variations, Dual molecular diagnosis, T-box transcription factor 4

## Abstract

**Background:**

Application of whole genome sequencing (WGS) enables identification of non-coding variants that play a phenotype-modifying role and are undetectable by exome sequencing. Recently, non-coding regulatory single nucleotide variants (SNVs) have been reported in patients with lethal lung developmental disorders (LLDDs) or congenital scoliosis with recurrent copy-number variant (CNV) deletions at 17q23.1q23.2 or 16p11.2, respectively.

**Case presentation:**

Here, we report a deceased newborn with pulmonary hypertension and pulmonary interstitial emphysema with features suggestive of pulmonary hypoplasia, resulting in respiratory failure and neonatal death soon after birth. Using the array comparative genomic hybridization and WGS*,* two heterozygous recurrent CNV deletions: ~ 2.2 Mb on 17q23.1q23.2, involving *TBX4*, and ~ 600 kb on 16p11.2, involving *TBX6,* that both arose de novo on maternal chromosomes were identified. In the predicted lung-specific enhancer upstream to *TBX4*, we have detected seven novel putative regulatory non-coding SNVs that were absent in 13 control individuals with the overlapping deletions but without any structural lung anomalies.

**Conclusions:**

Our findings further support a recently reported model of complex compound inheritance of LLDD in which both non-coding and coding heterozygous *TBX4* variants contribute to the lung phenotype. In addition, this is the first report of a patient with combined de novo heterozygous recurrent 17q23.1q23.2 and 16p11.2 CNV deletions.

## Background

Recurrent same-sized copy-number variant (CNV) deletions flanked by segmental duplications and mediated nonallelic homologous recombination (NAHR) are often identified in patients with different genomic disorders [1, 2]. A remarkable clinical heterogeneity and disease severity have been described among individuals with identical CNVs, suggesting that additional risk factors underlay the specific disease outcome. Analyses of the alleles unmasked by heterozygous deletions have enabled identification of both coding or non-coding phenotype-modifying variants, e.g. in patients with DiGeorge/Velocardiofacial/chromosome 22q11.2 deletion syndrome (MIM# 188400, 192430, and 611867) [3–5], Smith-Magenis syndrome (MIM# 182290) [6], thrombocytopenia-absent radius syndrome (MIM# 274000) [7], Prader–Willi (MIM# 176270) and Angelman (MIM# 105830) syndromes [8], chromosome 15q13.3 microdeletion syndrome (MIM# 612001) [9], chromosome 3q29 microdeletion syndrome (MIM# 609425) [10], neurofibromatosis type I (MIM# 162200) [11], and Sotos syndrome 1 (MIM# 117550) [12].

An extreme phenotypic variability has been reported in patients with a recurrent ~ 600 kb 16p11.2 CNV deletion, involving *TBX6* (MIM# 602427). This deletion has been associated with neurocognitive phenotypes, e.g. autism, epilepsy, and developmental delay, with coexisted congenital anomalies [13–16]. Interestingly, patients with this deletion have been found also to have an increased risk for neuroblastoma [17], cardiac defects [18], renal cysts [19], obesity [20], scoliosis, and vertebral anomalies [21–24]. Similar to 16p11.2 deletion, a recurrent 17q23.1q23.2 CNV deletion ~ 2.2 Mb in size and involving *TBX2* (MIM# 600747) and *TBX4* (MIM# 601719) has been reported in patients with a wide phenotypic heterogeneity. Ballif et al., 2010 described seven individuals with developmental delay, microcephaly, heart defects, limb abnormalities, and hearing loss [25]. Other patients with this deletion have also presented pulmonary arterial hypertension (PAH) and/or ischiocoxopodopatellar syndrome (MIM# 147891) [26–29]. Most recently, we and others have identified the heterozygous 17q23.1q23.2 CNV deletion in a series of individuals with lethal lung developmental disorders (LLDDs), including acinar dysplasia (AcDys), congenital alveolar dysplasia (CAD), and other forms of primary pulmonary hypoplasia (PH) [30–32]. The same lethal lung phenotypes were found in patients with heterozygous point mutations in *TBX4*, indicating it is the causative gene [31–33]. Interestingly, homozygous *TBX4* mutations manifest in lethal lung hypoplasia associated with multiple malformations, including complete posterior amelia with pelvis hypoplasia and heart defects [34, 35].

Here, we describe a deceased newborn with neonatal PAH and pulmonary interstitial emphysema with features suggestive of PH in whom molecular analyses revealed a de novo heterozygous recurrent CNV deletion on 17q23.1q23.2 with additional non-coding variants at the same locus, concomitant with a de novo heterozygous recurrent CNV deletion on 16p11.2.

## Case presentation

A female patient, born at 38 weeks’ gestation, was the first child of non-consanguineous parents. The pregnancy was uneventful and the amniotic fluid was noted to be of normal volume. Her birth weight was 3370 g and Apgar scores were 9 at 1 and 5 min. She was discharged to her mother but was found to be cyanotic at 5 hours of life and subsequently admitted to the neonatal intensive care unit (NICU). She required immediate intubation and ventilation. An echocardiogram showed a structurally normal heart but marked PAH. Ultrasound of the patient’s brain and abdomen was within normal limits. Her condition, however, deteriorated and she died within 14 h after admission to the NICU.

### Histopathological evaluation

**Fig. 1:**
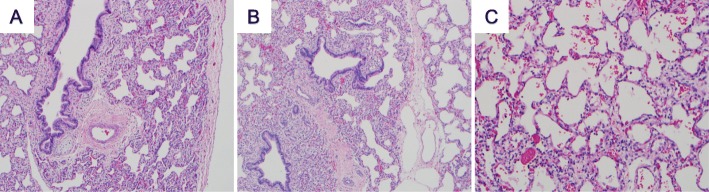
Histopathological characterization of the patient’s lungs. **a** Pulmonary hypoplasia; bronchus with cartilage is very close to the pleura, hematoxylin and eosin, (H&E), 4x. **b** Interlobular septum with dilated lymphatics and normally placed vein. Pulmonary arterioles are thickened. The respiratory bronchiole is too close to the septum, consistent with hypoplasia. H&E, 10x. **c** Mild growth disturbance with enlarged simplified alveoli, with normal capillaries

Histopathological evaluation was performed using formalin-fixed paraffin wax-embedded tissue from postmortem lung biopsies. Samples were examined by light microscopy using routine hematoxylin and eosin (H&E), Verhoeff’s van Gieson (EVG), periodic acid–Schiff–diastase (PAS-D), Perls’ Prussian blue and Masson’s trichrome stains. Post mortem lung biopsy of the right upper and middle lobes showed evidence of pulmonary hypertension and interstitial emphysema, with features suggestive of PH (Fig. 1a-c). On H&E staining, the general arrangement of the pulmonary arteries and veins was normal. The lung tissue, however, appeared hypoplastic with respiratory bronchioles noted very close to the pleural surface. The pulmonary arterial vessels were thick-walled, and there was a peripheral extension of smooth muscle into some of the alveolar septa, which were widened without increased cellularity or fibrosis. The interlobular septa were edematous, and there was marked lymphatic dilation and “hanging vessels”, consistent with pulmonary interstitial emphysema. The EVG, PAS-D, Perls’ Prussian blue, and Masson trichrome stains did not demonstrate any interstitial fibrosis or other abnormalities.

### Molecular analyses

Samples were collected from the proband (P094, lung tissue) and his parents (blood) after obtaining written informed consent. The study protocol was approved by the Institutional Review Board for Human Subject Research at Baylor College of Medicine (H-8712).

**Fig. 2:**
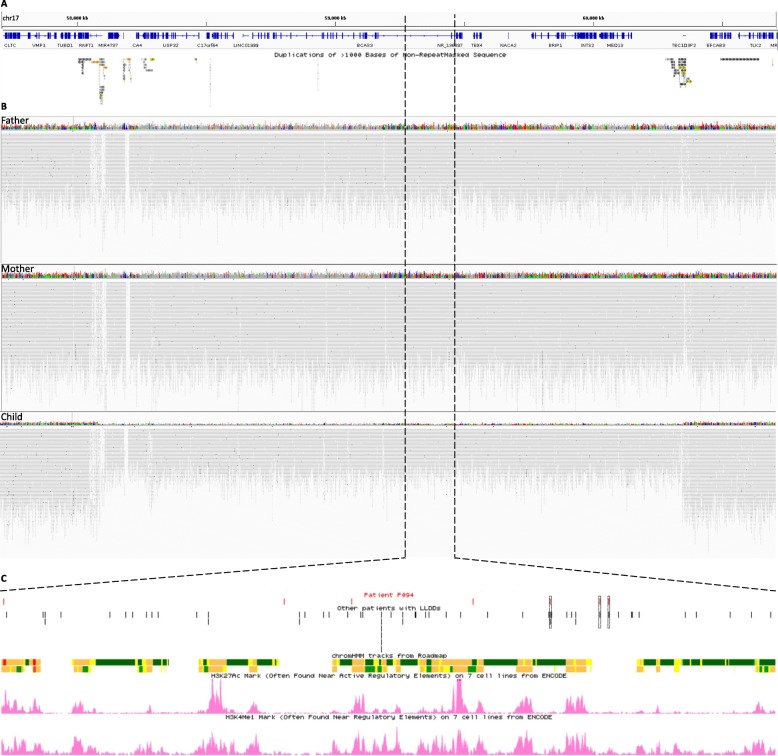
Schematic representation of 17q23.1q23.2 copy-number variant deletion region. **a** The 17q23.1q23.2 region (hg19) depicting the identified deletion in the presented patient with pulmonary hypoplasia. The genes mapping within the deletion, including *TBX4,* and complex low-copy repeats flanking the recurrent deletion are shown. **b** Alignment tracks showing whole genome sequencing coverage at 17q23.1q23.2 region in the father, mother, and child (upper, middle, and bottom track, respectively). **c** Distribution of single nucleotide variants (SNVs) in the putative lung-specific enhancer region located upstream to *TBX4*, identified in present subject (red) and other patients with lethal lung developmental disorders (black), are presented. Variants reported previously and also detected in present case are indicated by black dashed rectangles [31]. Chromatin state annotation track based on ChIP-seq mapping (Roadmap) in the IMR-90 cell line within the chr17:59,278,024-59,462,062 genomic region, as well as H3K27Ac, and H3K4Me1 marks found in fetal lung are shown below the SNVs track

Array comparative genomic hybridization (array CGH) was performed using proband’s DNA sample and a customized high-resolution 180 K microarray (Agilent Technologies, Santa Clara, CA, USA) with additional probes targeting genes involved in lung development, as described [31]. Whole genome sequencing (WGS) for the family trio was performed with a TruSeq Nano DNA HT Library Prep Kit (Illumina, San Diego, CA, USA) and the HiSeqX platform (Illumina) with mean coverage depth 30X at CloudHealth Genomics (Shanghai, China) and the data was processed according to previously described protocol [31]. Parental origin of the identified deletions was determined using informative single nucleotide variants (SNVs) from critical trio WGS analysis. Array CGH revealed two pathogenic de novo heterozygous recurrent CNV deletions: ~ 2.2 Mb on 17q23.1q23.2, involving *TBX4*, and ~ 0.6 Mb on 16p11.2, involving *TBX6*, both flanked by complex low-copy repeats. The probability of occurrence of these two CNV deletions in one individual is approximately 5e-10. A trio-based WGS analysis confirmed these findings (Fig. 2a, b, Additional file 1) and showed that they both arose on the maternal chromosomes (Additional file 2).

### Computational analysis

The enrichment of non-coding variants within and upstream to *TBX4* was analyzed using WGS data obtained from the presented newborn and the previously described cohort of eight patients with LLDD and 17q23.1q23.2 deletion as well as 13 control individuals with the same deletion but without any structural lung abnormalities [31]. Only variants with MAF < 10% (gnomAD r2.0.2) carried by at least two individuals with lung disease and absent in controls were considered in the analysis [31]. To test whether there is an excess of selected variants in a given region ***A***, a Monte Carlo approach was used. We estimated the empirical distribution of the number of variants selected in the previous step that fall into randomly selected genomic intervals of the fixed size (equal to the size of region ***A***) sampled from the 17q23.1q23.2 deletion region. *P*-value was calculated by dividing the number of intervals containing the same number or more variants than in the region ***A*** by the total number of sampled intervals.

We have observed enrichment of pre-selected non-coding variants mapping upstream (chr17:59,279,024-59,462,062, *p* = 0.0418) to *TBX4* present in cases and absent in controls when compared to the remainder of the deletion region (Additional file 3A, B). No clinically relevant coding SNVs have been found in the unmasked 17q23.1q23.2 deletion region in our patient. Examination of the interval mapping upstream to *TBX4* (chr17:59,278,024-59,462,062) that overlaps the predicted lung regulatory elements identified in human fetal lung fibroblasts (IMR-90), revealed seven non-coding SNVs: rs532804594, rs117665209, rs72277620, rs769013747, rs3785850, rs35383405, and rs143541906 (Fig. 2c, Table 1, Additional file 4) none of which were detected in 13 control subjects with the same CNV deletion but without any structural lung anomalies (Additional file 3A). Of note, three variants: rs3785850, rs35383405, and rs143541906 were previously identified in LLDD children (*n* = 2, *n* = 1, and *n* = 1) with 17q23.1q23.2 CNV deletion [31].

**Table 1:** Non-coding SNVs in the lung-specific enhancer region, identified in the newborn (P094) with 17q23.1q23.2 CNV deletion and lethal lung disease and absent in the control individuals with the same deletion but without lung abnormalities

Position [hg19]	rs^**a**^	Ref	Alt	MAF^**b**^	Alt allele count/ Allele number	Previous appearance (number of individuals)^**c**^
chr17:59278456–59,278,456	rs532804594	TAAGA	–	0.0008	24/31346	0
chr17:59345202–59,345,202	rs117665209	C	T	0.0167	527/31326	0
chr17:59361129–59,361,129	rs72277620	A	–	0.0142	443/31042	0
chr17:59390028–59,390,028	rs769013747	CTGGTTTCCATGCC	–	0.0003	9/31400	0
chr17:59408341–59,408,341	rs3785850	G	A	0.1219	3819/31378	2
chr17:59420152–59,420,152	rs35383405	G	T	0.1169	3/29938	1
chr17:59422277–59,422,277	rs143541906	T	TAC	0.0937	2862/30240	1

Abbreviations are as follows: +, present; −, absent; *Alt* altered allele, *MAF*, minor allele frequency, *NA* not applicable, *Ref* reference allele, ^a^rs numbers based on dbSNP v.150; ^b^MAF and allele number based on the GnomAD database (r2.0.2); ^c^Number of previously reported LLDD individuals with 17q23.1q23.2 CNV deletion and given non-coding variant

Analysis of a common haplotype defined by one synonymous SNV rs2289292 and two non-coding SNVs rs3809624 and rs3809627 in *TBX6,* previously associated with congenital scoliosis in up to 11% of Han Chinese with 16p11.2 deletion and present in 44% of Han Chinese, did not reveal its presence in our patient.

## Discussion and conclusion

TBX2, TBX4, and TBX6 are members of the T-box family transcription factors that are important regulators of embryonic development in vertebrates [36]. All T-box proteins share a conserved T-box motif interacting with specific DNA sequences to repress or activate transcription [36]. T-box genes are expressed in numerous tissues in a highly specific manner and mutations or CNVs containing T-box family members have been associated with different developmental disorders [37]. One of the most well characterized syndromes involving a T-box gene is DiGeorge/ Velocardiofacial/ chromosome 22q11.2 deletion syndrome caused by deletion of *TBX1* and characterized by congenital heart disease, immune deficiency, and developmental delay [3–5]. Other examples include ulnar-mammary syndrome (MIM# 181450) associated with *TBX3* mutations [38] or Holt-Oram syndrome caused by *TBX5* haploinsufficiency [39, 40]. Interestingly, there are also reports presenting overlapping features of these two syndromes in patients with contiguous deletion of both *TBX3* and *TBX5* [41]. *TBX2* abnormalities have been associated with a cardiovascular and skeletal developmental disorder [25, 42].

Recently, we and others have described heterozygous recurrent and nonrecurrent CNV deletions on 17q23.1q23.2, involving *TBX2* and *TBX4*, as well as de novo heterozygous missense *TBX4* variants [30–33] in patients with PH and other lethal pulmonary abnormal growth conditions. PH is a group of rare lung developmental diseases histopathologically characterized by a reduction of the number and size of bronchioles and alveoli [43, 44]. While PH is usually secondary to underlying disorders limiting fetal lung growth (i.e. diaphragmatic hernia, skeletal abnormalities, or oligohydramnios), primary PH (MIM# 265430) is related to an embryologic defect of lung branching morphogenesis and vasculogenesis [45, 46]. The consequence of PH is severe respiratory distress and PAH, typically refractory to therapy [44].

*TBX2* and *TBX4* are essential for normal development, including proper lung organogenesis [37]. Dysregulation of these genes in mice leads to a reduction of lung branching [47, 48], supporting the notion that 17q23.1q23.2 CNV deletions, detected in our newborn and other patients, are causative for their lethal lung phenotypes. Although SNVs and CNVs involving *TBX4* confer a risk of lung disease, the heterogeneity of clinical features associated with *TBX4* abnormalities suggests that they are not sufficient to lead to specific phenotypes and that lung phenotype cannot be explained by *TBX4* haploinsufficiency alone. We proposed a model of complex compound inheritance of LLDD [31]. Importantly, along with *TBX4* abnormalities, reported individuals with LLDDs were found to also have at least one rare or common non-coding SNV within an ~ 200 kb interval mapping ~ 70 kb upstream to *TBX4* and overlapping the predicted lung-specific enhancer [49], suggesting that this second risk allele with the putative hypomorphic variants *in trans* may affect *TBX4* and is required to cause a lethal lung disease [31]. In our patient, in this region, we have identified seven non-coding SNVs that are absent in 13 control subjects [31] with the same CNV deletion but without any structural lung anomalies. Notably, three of these variants: rs3785850, rs35383405, and rs143541906 were previously identified in LLDD children, making them better candidates [31]. However, the small size of our control group may be a limitation of this study.

To date, abnormalities involving three different T-box transcription factors have not been reported. It is unclear whether the 16p11.2 deletion contributed to the patient’s phenotype [14, 15]. Compound inheritance of 16p11.2 CNV deletion or coding SNV involving *TBX6* with the non-coding common T-C-A risk haplotype *in trans* has been associated with congenital vertebral malformations [22–24]. However, we did not find this non-coding haplotype in our patient and there was no evidence of congenital scoliosis or spondylocostal dysostosis that might have led to secondary pulmonary hypoplasia. Moreover, neither *TBX6* nor any other gene mapping within the 16p11.2 CNV deletion has been associated with lung development or function in humans. These data and the fact that 11 other patients with similar lethal lung developmental disorders and pathogenic heterozygous CNV deletions or SNVs involving *TBX4* [31] did not have any clinically relevant variants involving *TBX6* argue against the 16p11.2 CNV deletion contribution to the abnormal lung phenotype and two-hit hypothesis for CNVs proposed by Giriraian et al. [50].

Both CNV deletions in our patient arose de novo on maternal 17q23.1q23.2 and 16p11.2 chromosomes. While the maternal origin of the 16p11.2 CNV deletion in the presented child further confirms the findings that 89.4% of de novo 16p11.2 CNV deletions arose on the maternal chromosome 16 [51], there is an insufficient number of studied de novo 17q23.1q23.2 CNV deletions to conclude about their parental origin.

Multi-locus genomic variations and dual molecular diagnoses involving SNVs or CNVs have been increasingly described [52, 53]. While a combination of two different SNVs is the most commonly detected in patients with dual molecular diagnosis, the combination of CNVs and SNVs or two various CNVs have been rarely observed [52–54]. Examples of co-occurrence of two de novo CNVs include deletion of 22q11 and 10p14 in a patient with overlapping features of both 22q11 deletion syndrome and hypoparathyroidism, sensorineural deafness, and renal disease [55], 6q13q14.1 and 6q21q22.31 CNV deletions in a patient with Pierre Robin sequence and developmental delay [56] or recurrent CNV deletions of 7q11.23 and 22q11.2 in a patient with an unique phenotype and features specific for Williams and DiGeorge/ Velocardiofacial syndromes [57]. Analysis of a large cohort of children with CNV associated with intellectual disability and congenital abnormalities revealed the presence of a second CNV in 10.1% of studied individuals [58]. However, in the vast majority of these cases, at least one large CNV event was inherited from one of the parents [58].

In summary, we present the clinical and molecular findings in a newborn with PAH and pulmonary interstitial emphysema with features suggestive of PH, leading to respiratory failure and neonatal death on the first day of life in whom we detected de novo 17q23.1q23.2 and 16p11.2 CNV deletions. We have identified novel candidate regulatory SNVs in the potential lung-specific enhancer region mapping upstream to *TBX4*, as well as three variants previously detected in LLDD children patients. Our data further support the complex compound inheritance model for LLDDs due to a combination of rare coding variants involving *TBX4* with rare and common non-coding variants *in trans*.

## Supplementary information

**Additional file 1. **Schematic representation of 16p11.2 copy-number variant (CNV) deletion region. **A**) The 16p11.2 CNV region (hg19) depicting the identified deletion in the presented patient with pulmonary hypoplasia. The genes mapping within the deletion and complex low-copy repeats flanking the recurrent deletion are shown. **B**) Alignment tracks showing whole genome sequencing coverage at 16p11.2 CNV region in the father, mother, and child (upper, middle, and bottom track, respectively).

**Additional file 2.** The list of single nucleotide variants used for determination of the parental origin of 16p11.2 and 17q23.2 copy-number variant deletions.

**Additional file 3. **Distribution of the selected SNVs identified by whole genome sequencing in the 17q23.1q23.2 copy-number variant (CNV) deletion region (hg19) showing their enrichment. **A**) Enrichment of variants with minor allele frequency (MAF) < 10% (GnomAD, r2.0.2) observed in the presented patient (AD094). **B**) Enrichment of variants with MAF < 10% (GnomAD, r2.0.2) observed in the patient AD094 and previously reported patients with lethal lung developmental disorder and 17q23.1q23.2 CNV deletion.

**Additional file 4. **Non-coding single nucleotide variants in the lung-specific enhancer region, identified in newborns with 17q23.1q23.2 copy-number variant deletion or *TBX4* mutation and lethal lung disease and absent in the control individuals with the same deletion but without lung abnormalities.

## Data Availability

The datasets used and/or analyzed during the current study are available from the corresponding author on reasonable request.

## Abbreviations

AcDys: Acinar dysplasia
aCGH: array comparative genomic hybridization
CAD: Congenital alveolar dysplasia
CNV: Copy-number variant
EVG: Verhoeff’s van Gieson
H&E: Hematoxylin and eosin
LLDD: Lethal lung developmental disorder
NAHR: Nonallelic homologous recombination
NICU: Neonatal intensive care unit
PAH: Pulmonary arterial hypertension
PAS-D: Periodic acid–Schiff–diastase
PH: Pulmonary hypoplasia
SNV: Single nucleotide variant
WGS: Whole genome sequencing

## References

[1] Stankiewicz P, Lupski JR (2002). Genome architecture, rearrangements and genomic disorders. Trends Genet.

[2] Sharp AJ, Locke DP, McGrath SD, Cheng Z, Bailey JA, Vallente RU (2005). Segmental duplications and copy-number variation in the human genome. Am J Hum Genet.

[3] Perez E, Sullivan KE (2002). Chromosome 22q11.2 deletion syndrome (DiGeorge and velocardiofacial syndromes). Curr Opin Pediatr.

[4] Ben-Shachar S, Ou Z, Shaw CA, Belmont JW, Patel MS, Hummel M (2008). 22q11.2 distal deletion: a recurrent genomic disorder distinct from DiGeorge syndrome and velocardiofacial syndrome. Am J Hum Genet.

[5] McDonald-McGinn DM, Sullivan KE, Marino B, Philip N, Swillen A, Vorstman JAS (2015). 22q11.2 deletion syndrome. Nat Rev Dis Primers.

[6] Potocki L, Shaw CJ, Stankiewicz P, Lupski JR (2003). Variability in clinical phenotype despite common chromosomal deletion in Smith-Magenis syndrome [del(17)(p11.2p11.2)]. Genet Med.

[7] Albers CA, Paul DS, Schulze H, Freson K, Stephens JC, Smethurst PA (2012). Compound inheritance of a low-frequency regulatory SNP and a rare null mutation in exon-junction complex subunit RBM8A causes TAR syndrome. Nat Genet.

[8] Christian SL, Fantes JA, Mewborn SK, Huang B, Ledbetter DH (1999). Large genomic duplicons map to sites of instability in the Prader-Willi/Angelman syndrome chromosome region (15q11-q13). Hum Mol Genet.

[9] Ben-Shachar S, Lanpher B, German JR, Qasaymeh M, Potocki L, Nagamani SCS (2009). Microdeletion 15q13.3: a locus with incomplete penetrance for autism, mental retardation, and psychiatric disorders. J Med Genet.

[10] Willatt L, Cox J, Barber J, Cabanas ED, Collins A, Donnai D (2005). 3q29 microdeletion syndrome: clinical and molecular characterization of a new syndrome. Am J Hum Genet.

[11] Dorschner MO, Sybert VP, Weaver M, Pletcher BA, Stephens K (2000). NF1 microdeletion breakpoints are clustered at flanking repetitive sequences. Hum Mol Genet.

[12] Kurotaki N, Stankiewicz P, Wakui K, Niikawa N, Lupski JR (2005). Sotos syndrome common deletion is mediated by directly oriented subunits within inverted Sos-REP low-copy repeats. Hum Mol Genet.

[13] Weiss LA, Shen Y, Korn JM, Arking DE, Miller DT, Fossdal R (2008). Association between microdeletion and microduplication at 16p11.2 and autism. N Engl J Med.

[14] Shinawi M, Liu P, Kang S-HL, Shen J, Belmont JW, Scott DA (2010). Recurrent reciprocal 16p11.2 rearrangements associated with global developmental delay, behavioural problems, dysmorphism, epilepsy, and abnormal head size. J Med Genet.

[15] Rosenfeld JA, Coppinger J, Bejjani BA, Girirajan S, Eichler EE, Shaffer LG (2010). Speech delays and behavioral problems are the predominant features in individuals with developmental delays and 16p11.2 microdeletions and microduplications. J Neurodev Disord.

[16] Vlaskamp DRM, Callenbach PMC, Rump P, Giannini LAA, Brilstra EH, Dijkhuizen T (2019). *PRRT2*-related phenotypes in patients with a 16p11.2 deletion. Eur J Med Genet.

[17] Egolf LE, Vaksman Z, Lopez G, Rokita JL, Modi A, Basta PV (2019). Germline 16p11.2 Microdeletion Predisposes to Neuroblastoma. Am J Hum Genet.

[18] Xie H, Hong N, Zhang E, Li F, Sun K, Yu Y (2019). Identification of rare copy number variants associated with pulmonary atresia with ventricular Septal defect. Front Genet.

[19] Hernando C, Plaja A, Rigola MA, Pérez MM, Vendrell T, Egocue J (2002). Comparative genomic hybridisation shows a partial de novo deletion 16p11.2 in a neonate with multiple congenital malformations. J Med Genet.

[20] Walters RG, Jacquemont S, Valsesia A, de Smith AJ, Martinet D, Andersson J (2010). A new highly penetrant form of obesity due to deletions on chromosome 16p11.2. Nature.

[21] Al-Kateb H, Khanna G, Filges I, Hauser N, Grange DK, Shen J (2014). Scoliosis and vertebral anomalies: additional abnormal phenotypes associated with chromosome 16p11.2 rearrangement. Am J Med Genet A.

[22] Wu N, Ming X, Xiao J, Wu Z, Chen X, Shinawi M (2015). *TBX6* null variants and a common hypomorphic allele in congenital scoliosis. N Engl J Med.

[23] Yang N, Wu N, Zhang L, Zhao Y, Liu J, Liang X (2018). *TBX6* compound inheritance leads to congenital vertebral malformations in humans and mice. Hum Mol Genet.

[24] Liu J, Wu N, Yang N, Takeda K, Chen W, Deciphering Disorders Involving Scoliosis and COmorbidities (DISCO) study (2019). TBX6-associated congenital scoliosis (TACS) as a clinically distinguishable subtype of congenital scoliosis: further evidence supporting the compound inheritance and *TBX6* gene dosage model. Genet Med.

[25] Ballif BC, Theisen A, Rosenfeld JA, Traylor RN, Gastier-Foster J, Thrush DL (2010). Identification of a recurrent microdeletion at 17q23.1q23.2 flanked by segmental duplications associated with heart defects and limb abnormalities. Am J Hum Genet.

[26] Nimmakayalu M, Major H, Sheffield V, Solomon DH, Smith RJ, Patil SR (2011). Microdeletion of 17q22q23.2 encompassing *TBX2* and *TBX4* in a patient with congenital microcephaly, thyroid duct cyst, sensorineural hearing loss, and pulmonary hypertension. Am J Med Genet A.

[27] Kerstjens-Frederikse WS, Bongers EMHF, Roofthooft MTR, Leter EM, Douwes JM, Van Dijk A (2013). *TBX4* mutations (small patella syndrome) are associated with childhood-onset pulmonary arterial hypertension. J Med Genet.

[28] Galambos C, Mullen MP, Shieh JT, Schwerk N, Kielt MJ, Ullmann N (2019). Phenotype characterisation of *TBX4* mutation and deletion carriers with neonatal and paediatric pulmonary hypertension. Eur Respir J.

[29] Maurac A, Lardenois É, Eyries M, Ghigna MR, Petit I, Montani D (2019). T-box protein 4 mutation causing pulmonary arterial hypertension and lung disease. Eur Respir J.

[30] German K, Deutsch GH, Freed AS, Dipple KM, Chabra S, Bennett JT (2019). Identification of a deletion containing *TBX4* in a neonate with acinar dysplasia by rapid exome sequencing. Am J Med Genet A.

[31] Karolak JA, Vincent M, Deutsch G, Gambin T, Cogné B, Pichon O (2019). Complex compound inheritance of lethal lung developmental disorders due to disruption of the TBX-FGF pathway. Am J Hum Genet.

[32] Suhrie K, Pajor NM, Ahlfeld SK, Dawson DB, Dufendach KR, Kitzmiller JA (2018). Neonatal lung disease associated with *TBX4* mutations. J Pediatr.

[33] Szafranski P, Coban-Akdemir ZH, Rupps R, Grazioli S, Wensley D, Jhangiani SN (2016). Phenotypic expansion of *TBX4* mutations to include acinar dysplasia of the lungs. Am J Med Genet A.

[34] Kariminejad A, Szenker-Ravi E, Lekszas C, Tajsharghi H, Moslemi A-R, Naert T (2019). Homozygous null *TBX4* mutations Lead to posterior Amelia with pelvic and pulmonary hypoplasia. Am J Hum Genet.

[35] Ranganath P, Perala S, Nair L, Pamu PK, Shankar A, Murugan S, et al. A newly recognized multiple malformation syndrome with caudal regression associated with a biallelic c.402G>A variant in *TBX4*. Eur J Hum Genet. 2020. 10.1038/s41431-020-0572-5. 10.1038/s41431-020-0572-5 PMC7170885 31965066

[36] Agulnik SI, Garvey N, Hancock S, Ruvinsky I, Chapman DL, Agulnik I (1996). Evolution of mouse T-box genes by tandem duplication and cluster dispersion. Genetics.

[37] Papaioannou VE (2014). The T-box gene family: emerging roles in development, stem cells and cancer. Development.

[38] Bamshad M, Lin RC, Law DJ, Watkins WC, Krakowiak PA, Moore ME (1997). Mutations in human *TBX3* alter limb, apocrine and genital development in ulnar-mammary syndrome. Nat Genet.

[39] Li QY, Newbury-Ecob RA, Terrett JA, Wilson DI, Curtis AR, Yi CH (1997). Holt-Oram syndrome is caused by mutations in *TBX5*, a member of the Brachyury (T) gene family. Nat Genet.

[40] Basson CT, Bachinsky DR, Lin RC, Levi T, Elkins JA, Soults J (1997). Mutations in human *TBX5* cause limb and cardiac malformation in Holt-Oram syndrome. Nat Genet.

[41] Borozdin W, Bravo-Ferrer Acosta AM, Seemanova E, Leipoldt M, Bamshad MJ, Unger S (2006). Contiguous hemizygous deletion of *TBX5*, *TBX3*, and *RBM19* resulting in a combined phenotype of Holt-Oram and ulnar-mammary syndromes. Am J Med Genet A.

[42] Liu N, Schoch K, Luo X, Pena LDM, Bhavana VH, Kukolich MK (2018). Functional variants in *TBX2* are associated with a syndromic cardiovascular and skeletal developmental disorder. Hum Mol Genet.

[43] Porter HJ (1999). Pulmonary hypoplasia. Arch Dis Child Fetal Neonatal Ed.

[44] Vincent M, Karolak JA, Deutsch G, Gambin T, Popek E, Isidor B (2019). Clinical, Histopathological, and molecular diagnostics in lethal lung developmental disorders. Am J Respir Crit Care Med.

[45] Swischuk LE, Richardson CJ, Nichols MM, Ingman MJ (1979). Primary pulmonary hypoplasia in the neonate. J Pediatr.

[46] Odd DE, Battin MR, Hallam L, Knight DB (2003). Primary pulmonary hypoplasia: a case report and review of the literature. J Paediatr Child Health.

[47] Arora R, Metzger RJ, Papaioannou VE (2012). Multiple roles and interactions of *Tbx4* and *Tbx5* in development of the respiratory system. PLoS Genet.

[48] Lüdtke TH, Rudat C, Wojahn I, Weiss A-C, Kleppa M-J, Kurz J (2016). *Tbx2* and *Tbx3* Act Downstream of Shh to Maintain Canonical Wnt Signaling during Branching Morphogenesis of the Murine Lung. Dev Cell.

[49] Hnisz D, Abraham BJ, Lee TI, Lau A, Saint-André V, Sigova AA (2013). Super-enhancers in the control of cell identity and disease. Cell.

[50] Girirajan S, Rosenfeld JA, Cooper GM, Antonacci F, Siswara P, Itsara A (2010). A recurrent 16p12.1 microdeletion supports a two-hit model for severe developmental delay. Nat Genet.

[51] Duyzend MH, Nuttle X, Coe BP, Baker C, Nickerson DA, Bernier R (2016). Maternal modifiers and parent-of-origin Bias of the autism-associated 16p11.2 CNV. Am J Hum Genet.

[52] Posey JE, Harel T, Liu P, Rosenfeld JA, James RA, Coban Akdemir ZH (2017). Resolution of Disease Phenotypes Resulting from Multilocus Genomic Variation. N Engl J Med.

[53] Dharmadhikari AV, Ghosh R, Yuan B, Liu P, Dai H, Al Masri S (2019). Copy number variant and runs of homozygosity detection by microarrays enabled more precise molecular diagnoses in 11,020 clinical exome cases. Genome Med.

[54] Liu P, Yuan B, Carvalho CMB, Wuster A, Walter K, Zhang L (2017). An organismal CNV mutator phenotype restricted to early human development. Cell.

[55] Fukai R, Ochi N, Murakami A, Nakashima M, Tsurusaki Y, Saitsu H (2013). Co-occurrence of 22q11 deletion syndrome and HDR syndrome. Am J Med Genet A.

[56] Parmeggiani G, Bigoni S, Buldrini B, Garani G, Clauser L, Galiè M (2017). Double interstitial deletion of the long arm of chromosome 6 in a patient with Pierre Robin sequence, Dysmorphisms, and severe developmental delay. Mol Syndromol.

[57] Shukla A, Mandal K, Patil SJ, Kishore Y, Phadke SR, Girisha KM (2015). Co-occurrence of a de novo Williams and 22q11.2 microdeletion syndromes. Am J Med Genet A.

[58] Girirajan S, Rosenfeld JA, Coe BP, Parikh S, Friedman N, Goldstein A (2012). Phenotypic heterogeneity of genomic disorders and rare copy-number variants. N Engl J Med.

