# Supplementary material for: A de novo 2.2 Mb recurrent 17q23.1q23.2 deletion unmasks novel putative regulatory non-coding SNVs associated with lethal lung hypoplasia and pulmonary hypertension: a case report
Source: BMC Med Genomics. 2020 Mar 6;13:34. doi: 10.1186/s12920-020-0701-6 (PMC7060516; doi:10.1186/s12920-020-0701-6)
Supplement: Supplementary file 1 — Additional file 1. Schematic representation of 16p11.2 copy-number variant (CNV) deletion region. A) The 16p11.2 CNV region (hg19) depicting the identified deletion in the presented patient with pulmonary hypoplasia. The genes mapping within the deletion and complex low-copy repeats flanking the recurrent deletion are shown. B) Alignment tracks showing whole genome sequencing coverage at 16p11.2 CNV region in the father, mother, and child (upper, middle, and bottom track, respectively). [file 12920_2020_701_MOESM1_ESM.docx]

**ADDITIONAL FILES**

**Additional file 1.** Schematic representation of 16p11.2 CNV deletion region. **A**) The 16p11.2 CNV region (hg19) depicting the identified deletion in the presented patient with pulmonary hypoplasia. The genes mapping within the deletion and complex low-copy repeats flanking the recurrent deletion are shown. **B**) Alignment tracks showing whole genome sequencing coverage at 16p11.2 CNV region in the father, mother, and child (upper, middle, and bottom track, respectively).
