# Supplementary material for: A de novo 2.2 Mb recurrent 17q23.1q23.2 deletion unmasks novel putative regulatory non-coding SNVs associated with lethal lung hypoplasia and pulmonary hypertension: a case report
Source: BMC Med Genomics. 2020 Mar 6;13:34. doi: 10.1186/s12920-020-0701-6 (PMC7060516; doi:10.1186/s12920-020-0701-6)
Supplement: Supplementary file 2 — Additional file 2. The list of single nucleotide variants used for determination of the parental origin of 16p11.2 and 17q23.2 copy-number variant deletions. [file 12920_2020_701_MOESM2_ESM.docx]

**Additional file 2**. List of single nucleotide variants used for determination of parental origin of 16p11.2 and 17q23.2 copy number variant (CNV) deletions.

| Position [hg19] | Father | Mother | Child | Parent of origin of CNV deletion |
| --- | --- | --- | --- | --- |
| chr16:30043110 | Total count: 36 A : 0 C : 0 **G : 36 (100%, 24+, 12-)** T : 0 | Total count: 41 A : 0 C : 0 **G : 0** T : 41 (100%, 14+, 27-) | Total count: 14 A : 0 C : 0 **G : 14 (100%, 6+, 8-)** T : 0 | Mother |
| chr16:30079724 | Total count: 33 A : 0 **C : 21 (64%, 14+, 7-)** G : 0 T : 12 (36%, 6+, 6-) | Total count: 36 A : 0 **C : 0** G : 0 T : 36 (100%, 25+, 11-) | Total count: 13 A : 0 **C : 13 (100%, 6+, 7-)** G : 0 T : 0 | Mother |
| chr16:30095492 | Total count: 40 A : 0 C : 21 (53%, 11+, 10-) G : 0 **T : 19 (48%, 5+, 14-)** | Total count: 23 A : 0 C : 23 (100%, 12+, 11-) G : 0 **T : 0** | Total count: 28 A : 0 C : 0 G : 0 **T : 28 (100%, 11+, 17-)** | Mother |
| chr16:30100789 | Total count: 46 A : 0 **C : 26 (57%, 16+, 10-)** G : 0 T : 20 (43%, 10+, 10-) | Total count: 42 A : 0 **C : 0** G : 0 T : 42 (100%, 15+, 27-) | Total count: 17 A : 0 **C : 17 (100%, 13+, 4-)** G : 0 T : 0 | Mother |
| chr16:30101596 | Total count: 39 **A : 39 (100%, 24+, 15-)** C : 0 G : 0 T : 0 | Total count: 49 **A : 0** C : 49 (100%, 28+, 21-) G : 0 T : 0 | Total count: 28 **A : 28 (100%, 17+, 11-)** C : 0 G : 0 T : 0 | Mother |
| chr17:59328138 | Total count: 30 A : 0 C : 17 (57%, 1+, 16-) G : 0 **T : 13 (43%, 7+, 6-)** | Total count: 51 A : 0 C : 51 (100%, 37+, 14-) G : 0 **T : 0** | Total count: 25 A : 0 C : 0 G : 0 **T : 25 (100%, 10+, 15-)** | Mother |
| chr17:59478644 | Total count: 34 **A : 34 (100%, 17+, 17-)** C : 0 G : 0 T : 0 | Total count: 41 **A : 0** C : 0 G : 41 (100%, 14+, 27-) T : 0 | Total count: 14 **A : 14 (100%, 7+, 7-)** C : 0 G : 0 T : 0 | Mother |
| chr17:59545329 | Total count: 25 **A : 25 (100%, 9+, 16-)** C : 0 G : 0 T : 0 | Total count: 35 **A : 0** C : 35 (100%, 21+, 14-) G : 0 T : 0 | Total count: 10 **A : 10 (100%, 4+, 6-)** C : 0 G : 0 T : 0 | Mother |
| chr17:59559665 | Total count: 30 A : 0 C : 0 **G : 30 (100%, 21+, 9-)** T : 0 | Total count: 49 A : 0 C : 0 **G : 0** T : 49 (100%, 27+, 22-) | Total count: 19 A : 0 C : 0 **G : 19 (100%, 13+, 6-)** T : 0 | Mother |
| chr17:59767407 | Total count: 42 A : 0 **C : 42 (100%, 18+, 24-**) G : 0 T : 0 | Total count: 48 A : 0 **C : 0** G : 0 T : 48 (100%, 19+, 29-) | Total count: 21 A : 0 **C : 21 (100%, 13+, 8-)** G : 0 T : 0 | Mother |

Total count - total number of reads at particular position. Numbers of reads for every nucleotide at particular position are presented for each family member. Numbers in parentheses are as follows: “%” - percentage of reads for particular nucleotide, “+” number of reads for particular nucleotide; plus strand, “-” number of reads for particular nucleotide; minus strand.
