# Supplementary material for: A de novo 2.2 Mb recurrent 17q23.1q23.2 deletion unmasks novel putative regulatory non-coding SNVs associated with lethal lung hypoplasia and pulmonary hypertension: a case report
Source: BMC Med Genomics. 2020 Mar 6;13:34. doi: 10.1186/s12920-020-0701-6 (PMC7060516; doi:10.1186/s12920-020-0701-6)
Supplement: Supplementary file 3 — Additional file 3. Distribution of the selected SNVs identified by whole genome sequencing in the 17q23.1q23.2 copy-number variant (CNV) deletion region (hg19) showing their enrichment. A) Enrichment of variants with minor allele frequency (MAF) < 10% (GnomAD, r2.0.2) observed in the presented patient (AD094). B) Enrichment of variants with MAF < 10% (GnomAD, r2.0.2) observed in the patient AD094 and previously reported patients with lethal lung developmental disorder and 17q23.1q23.2 CNV deletion. [file 12920_2020_701_MOESM3_ESM.docx]

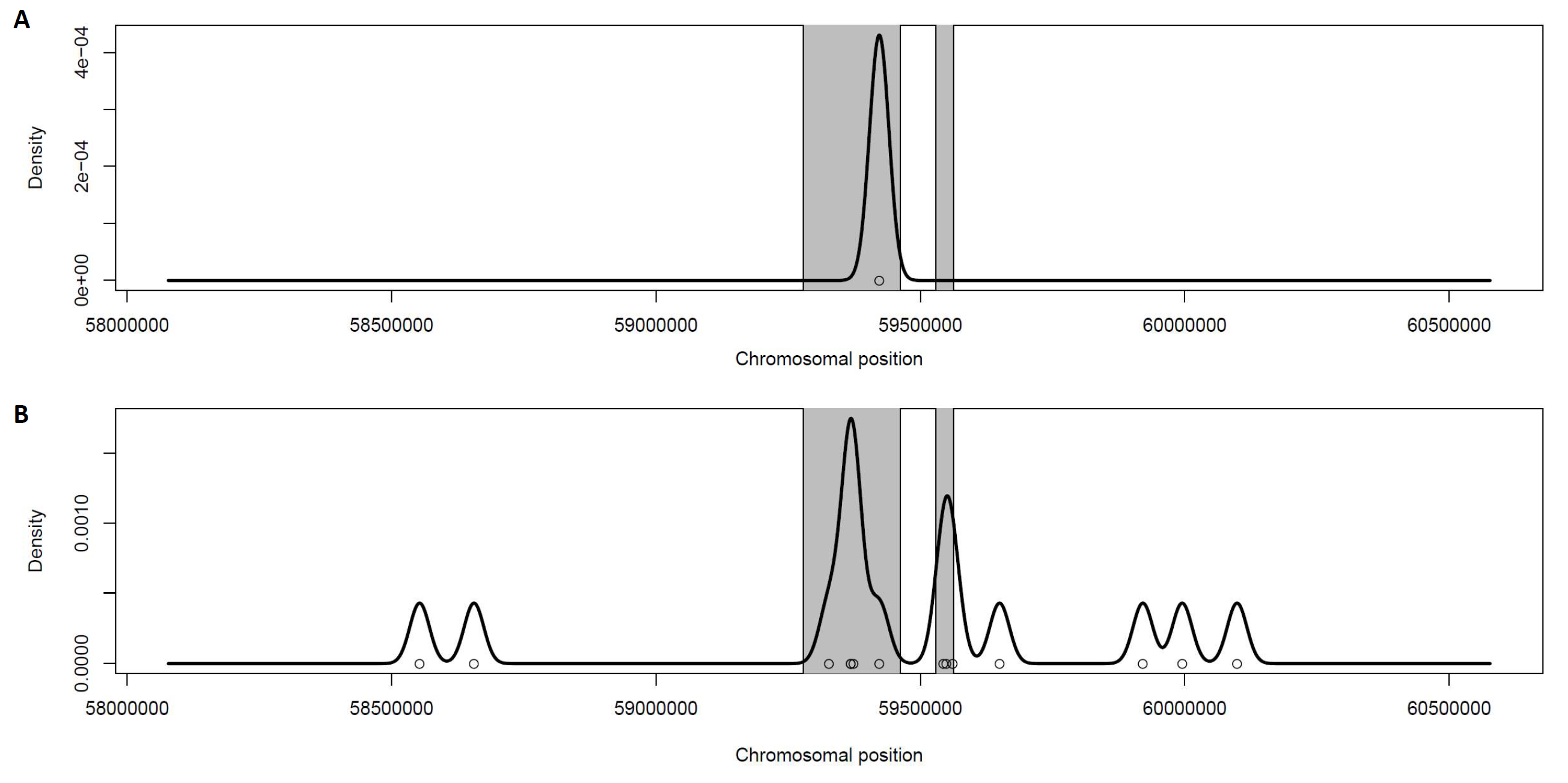


**Additional file 3.** Distribution of the selected SNVs identified by WGS in the 17q23.1q23.2 CNV deletion region (hg19) showing their enrichment. **A**) Enrichment of variants with MAF < 10% (GnomAD, r2.0.2) observed in presented patient (AD094). **B**) Enrichment of variants with MAF < 10% (GnomAD, r2.0.2) observed in patient AD094 and previously reported patients with lethal lung developmental disorder and 17q23.1q23.2 CNV deletion (Karolak et al., 2019).
