# Supplementary material for: A de novo 2.2 Mb recurrent 17q23.1q23.2 deletion unmasks novel putative regulatory non-coding SNVs associated with lethal lung hypoplasia and pulmonary hypertension: a case report
Source: BMC Med Genomics. 2020 Mar 6;13:34. doi: 10.1186/s12920-020-0701-6 (PMC7060516; doi:10.1186/s12920-020-0701-6)
Supplement: Supplementary file 4 — Additional file 4. Non-coding single nucleotide variants in the lung-specific enhancer region, identified in newborns with 17q23.1q23.2 copy-number variant deletion or TBX4 mutation and lethal lung disease and absent in the control individuals with the same deletion but without lung abnormalities. [file 12920_2020_701_MOESM4_ESM.docx]

**Additional file 4**. Non-coding SNVs in the lung-specific enhancer region identified in newborns with 17q23.1q23.2 CNV deletion or *TBX4* mutation and lethal lung disease and absent in the control individuals with the same deletion but without lung abnormalities (Karolak et al., 2019).

| **Position [hg19]** | **rs^a^** | **Ref** | **Alt** | **MAF^b^** | **Alt allele count/Allele number^b^** | **P006** | **P009** | **P012** | **P015/16** | **P019** | **P022** | **P025** | **P026** | **P035** | **P073** | **P094** |
| --- | --- | --- | --- | --- | --- | --- | --- | --- | --- | --- | --- | --- | --- | --- | --- | --- |
| chr17:59278456-59278456 | 532804594 | TAAGA | - | 0.0008 | 24/31346 | - | - | - | - | - | - | - | - | - | - | + |
| chr17:59279120-59279120 | NA | C | CTT | NA | NA | - | - | - | - | - | - | - | - | - | + | - |
| chr17:59287811-59287811 | 145662401 | G | T | 0.0039 | 124/31398 | + | - | - | - | - | - | - | - | - | - | - |
| chr17:59288406-59288406 | 8070692 | T | G | 0.2246 | 6985/31242 | - | + | - | - | - | - | - | - | - | + | - |
| chr17:59292085-59292085 | 117188060 | C | A | 0.0063 | 190/31350 | - | - | - | - | - | - | + | - | - | - | - |
| chr17:59303786-59303786 | 139983813 | G | A | 0.0051 | 163/31360 | + | - | - | - | - | - | - | - | - | - | - |
| chr17:59307503-59307503 | NA | T | TACAC | NA | NA | - | - | - | - | - | - | - | - | - | + | - |
| chr17:59309085-59309085 | 72832589 | T | C | 0.081 | 2511/31042 | - | + | - | - | - | - | - | - | - | - | - |
| chr17:59312457-59312457 | 138660616 | G | A | 0.0106 | 346/31366 | - | - | - | - | - | - | - | - | - | + | - |
| chr17:59313654-59313654 | 150043642 | T | C | 0.0003 | 10/31398 | - | - | - | - | - | - | + | - | - | - | - |
| chr17:59315155-59315155 | 940861097 | A | G | NA | NA | - | - | - | - | - | + | - | - | - | - | - |
| chr17:59324435-59324435 | 753135645 | C | T | 0.0002 | 7/31402 | - | - | - | - | - | - | - | - | - | + | - |
| chr17:59327165-59327165 | 35636245 | G | GA | 0.0407 | 1299/31254 | - | - | + | - | - | - | - | + | - | - | - |
| chr17:59345202-59345202 | 117665209 | C | T | 0.0167 | 527/31326 | - | - | - | - | - | - | - | - | - | - | + |
| chr17:59348785-59348785 | NA | A | ATTTTTTTTTTTTTTT | NA | NA | + | - | - | - | - | - | - | - | - | + | - |
| chr17:59349997-59349997 | NA | C | CAAAA | NA | NA | - | - | - | - | - | - | - | - | + | - | - |
| chr17:59354561-59354561 | 75380888 | T | C | 0.0248 | 782/30766 | - | - | - | - | - | - | - | + | - | - | - |
| chr17:59355734-59355734 | 567208829 | G | A | 0.0027 | 85/31366 | - | + | - | - | - | - | - | - | - | - | - |
| chr17:59360179-59360179 | 146403465 | T | C | 0.0217 | 683/31356 | - | - | - | - | - | + | - | - | - | - | - |
| chr17:59361129-59361129 | 72277620 | A | - | 0.0142 | 443/31042 | - | - | - | - | - | - | - | - | - | - | + |
| chr17:59363288-59363288 | 117484839 | C | T | 0.0063 | 196/31414 | + | - | - | - | - | - | - | - | - | - | - |
| chr17:59363880-59363880 | 117798644 | G | A | 0.0072 | 232/31390 | - | - | - | + | - | - | - | - | - | - | - |
| chr17:59368180-59368180 | 35827636 | T | C | 0.0793 | 4/31342 | + | - | + | - | - | + | - | - | + | - | - |
| chr17:59368293-59368293 | 192153557 | C | A | 0.0347 | 1094/31360 | - | + | - | - | - | - | - | - | - | + | - |
| chr17:59370539-59370539 | 112164816 | A | T | 0.0102 | 320/31406 | - | - | - | - | - | - | - | - | - | + | - |
| chr17:59373345-59373345 | 148383088 | C | T | 0.0194 | 598/31098 | + | - | - | - | - | + | - | - | - | - | - |
| chr17:59376344-59376344 | 139134582 | C | T | 0.002 | 60/31208 | - | - | - | - | - | - | + | - | - | - | - |
| chr17:59376380-59376380 | 561102192 | C | T | 0.0003 | 10/31110 | - | + | - | - | - | - | - | - | - | - | - |
| chr17:59378757-59378757 | 34867966 | G | A | 0.1443 | 4544/31358 | - | - | - | - | - | - | - | - | + | - | - |
| chr17:59379480-59379480 | NA | T | C | NA | NA | - | - | - | - | - | - | - | - | + | - | - |
| chr17:59383687-59383687 | 117259668 | G | A | 0.0107 | 342/31396 | - | - | - | + | - | - | - | - | - | - | - |
| chr17:59387086-59387086 | 973627683 | G | A | 0.0001 | 4/31384 | - | - | - | - | - | + | - | - | - | - | - |
| chr17:59390028-59390028 | 769013747 | CTGGTTTCCATGCC | - | 0.0003 | 9/31400 | - | - | - | - | - | - | - | - | - | - | + |
| chr17:59393463-59393463 | NA | C | T | NA | NA | - | - | - | - | - | - | - | - | - | + | - |
| chr17:59401781-59401781 | 117993484 | G | A | 0.0076 | 240/31398 | - | - | - | + | - | - | - | - | - | - | - |
| chr17:59408027-59408027 | 113520216 | C | T | 0.0102 | 319/31402 | - | - | - | - | - | - | - | - | - | + | - |
| chr17:59408341-59408341 | 3785850 | G | A | 0.1219 | 3819/31378 | - | - | - | - | - | - | + | - | - | + | + |
| chr17:59408765-59408765 | 190888982 | G | C | NA | NA | - | - | - | - | - | - | + | - | - | - | - |
| chr17:59412341-59412341 | 117088470 | C | T | 0.0069 | 206/31396 | - | - | - | - | - | - | - | - | + | - | - |
| chr17:59413482-59413482 | 7224107 | C | T | 0.1016 | 3182/31402 | - | - | - | - | - | - | - | + | - | - | - |
| chr17:59414473-59414473 | 566255513 | C | CAA | 0.1022 | 30124/29632 | - | - | - | - | - | - | - | + | + | - | - |
| chr17:59420152-59420152 | 35383405 | G | T | 0.1169 | 3/29938 | - | - | - | - | - | - | + | - | - | - | + |
| chr17:59422277-59422277 | 143541906 | T | TAC | 0.0937 | 2862/30240 | - | - | - | - | - | - | - | - | - | + | + |
| chr17:59424604-59424604 | 143968095 | G | A | 0.0662 | 2073/31356 | - | - | + | - | - | - | - | - | - | - | - |
| chr17:59427643-59427643 | 75073226 | G | A | 0.1128 | 3501/31210 | - | - | + | - | - | - | - | - | - | - | - |
| chr17:59427829-59427829 | 116271272 | G | A | 0.1074 | 3200/30012 | - | - | + | - | - | - | - | - | - | - | - |
| chr17:59429503-59429503 | 79390380 | G | A | 0.0741 | 2321/31380 | - | - | + | - | - | - | - | - | - | - | - |
| chr17:59440490-59440490 | 918478913 | G | A | NA | NA | - | + | - | - | - | - | - | - | - | - | - |
| chr17:59442994-59442994 | 116842887 | C | T | 0.0078 | 244/31372 | - | - | - | - | - | - | - | - | + | - | - |
| chr17:59451090-59451090 | NA | G | GCCCCC | NA | NA | - | - | + | - | - | - | - | - | - | - | - |
| chr17:59456218-59456218 | 80207525 | C | T | 0.0019 | 56/31406 | - | - | - | - | - | - | + | - | - | - | - |
| chr17:59460811-59460811 | 188999860 | G | C | 0.0001 | 3/31372 | - | - | - | - | - | - | + | - | - | - | - |
| chr17:59462062-59462062 | 117518238 | C | T | 0.018 | 568/31374 | - | - | - | - | + | - | - | - | - | - | - |

Abbreviations are as follows: +, present; -, absent; Alt, altered allele; MAF, minor allele frequency; NA, not applicable; Ref, reference allele; ^a^rs numbers based on dbSNP v.150; ^b^MAF and allele number based on the GnomAD database (r2.0.2)

**REFERENCES:**

Karolak, J. A., Vincent, M., Deutsch, G., Gambin, T., Cogné, B., Pichon, O., … Stankiewicz, P. (2019). Complex Compound Inheritance of Lethal Lung Developmental Disorders Due to Disruption of the TBX-FGF Pathway. *American Journal of Human Genetics*, *104*(2), 213–228.
